# Effects of Dietary Betaine on Growth Performance, Digestive Function, Carcass Traits, and Meat Quality in Indigenous Yellow-Feathered Broilers under Long-Term Heat Stress

**DOI:** 10.3390/ani9080506

**Published:** 2019-07-31

**Authors:** Wenchao Liu, Yilin Yuan, Chenyu Sun, Balamuralikrishnan Balasubramanian, Zhihui Zhao, Lilong An

**Affiliations:** 1 Department of Animal Science, College of Agriculture, Guangdong Ocean University, Zhanjiang 524088, China; 2 Department of Food Science and Biotechnology, College of Life Science, Sejong University, Seoul 05006, Korea

**Keywords:** broilers, digestive function, heat stress, indigenous yellow-feathered breed

## Abstract

**Simple Summary:**

Heat stress, one of the major problems in tropical and subtropical regions, adversely affects poultry production. This study was designed to evaluate the effects of dietary betaine on growth performance, digestive function, carcass traits, and meat quality in indigenous yellow-feathered broilers subjected to long-term heat stress. The results demonstrated that long-term heat exposure reduced the growth performance, digestive function, and carcass yield, and dietary betaine supplementation partially alleviated the adverse effects of heat stress on these parameters. These findings are useful for development of anti-heat stress feed additives in indigenous yellow-feathered broilers.

**Abstract:**

Heat stress has a profound effect on poultry health and productivity. The present study evaluated whether feeding betaine could ameliorate long-term heat stress-induced impairment of productive performance in indigenous yellow-feathered broilers. A total of 240 five-week-old male broilers were randomly allocated to five treatments with six replicates of eight broilers each. The five treatments included a thermoneutral zone control group (TN, fed basal diet), a heat stress control group (HS, fed basal diet), and an HS control group supplemented 500, 1000, 2000 mg/kg betaine, respectively. The TN group was raised at 26 ± 1 °C during the whole study, HS groups exposed to 32 ± 1 °C for 8 h/day from 9:00 am to 17:00 pm. The results showed that heat stress decreased the body weight gain (BWG) and feed intake of broilers during 1–5, 6–10, and 1–10 weeks (*p* < 0.05). Dietary betaine tended to improve the BWG and feed intake of broilers under 5 weeks of heat stress (linear, *p* < 0.10), and betaine supplementation linearly increased the BWG and feed intake during 6–10 and 1–10 weeks (*p* < 0.05). Additionally, nitrogen retention was reduced by 5 weeks and 10 weeks of heat stress (*p* < 0.05), whereas dietary betaine could improve nitrogen retention in heat stressed broilers after both 5 and 10 weeks of heat stress (linear, *p* < 0.05). Moreover, this study observed that the trypsin activity of jejunum was decreased by 5 weeks of heat stress (*p* < 0.05), whereas betaine supplementation had quadratic effects on trypsin activity of jejunum in heat stressed broilers (*p* < 0.05). Furthermore, 10 weeks of heat stress induced a reduction of villus height of the duodenum and jejunum (*p* < 0.05), and decreased the villus height to crypt depth ratio of the jejunum (*p* < 0.05). Supplementation with betaine ameliorated the adverse effects of heat stress on these parameters (*p* < 0.05). Compared with the TN group, 10 weeks of heat stress reduced carcass and breast yield (*p* < 0.05) and betaine supplementation improved carcass and breast yield of heat stressed broilers (linear, *p* < 0.05). In conclusion, dietary supplementation of betaine could reduce the detrimental effects of long-term heat stress on growth performance, digestive function, and carcass traits in indigenous yellow-feathered broilers.

## 1. Introduction

With global warming, the deleterious effects of heat stress induced by high ambient temperature on poultry productivity have been of great concern all over the world, especially in tropical and subtropical regions. Heat stress has a profound effect on broilers’ health and production, and leads to multiple physiological disturbances, such as endocrine disorders, systemic immune dysregulation, and electrolyte imbalance [1]. Heat stress also causes a disruption in the intestinal structure and function, including reduced regeneration and integrity of the intestinal epithelium [2,3], which in turn suppresses the growth rate and feed efficiency of birds. In addition, heat stress impairs carcass traits and meat quality through affecting energy-substance metabolism and redox status, resulting in decreased meat yield and increased abdominal fat rate in broilers [4,5]. Reducing the house temperature to the thermoneutral zone is a direct strategy to eliminate heat stress of poultry, and the thermoneutral zone can maximize the growth potential [1]. However, the cost of cooling equipment is relatively high in broiler production. It has previously been reported that nutritional manipulation could be a viable option to minimize the adverse impacts of heat stress on broilers [6], including supplementation of functional feed additives, such as probiotics, prebiotics, and natural active substances.

In recent years, special attention has been paid to the use of natural plant extracts in animal science. Betaine is a trimethyl derivative of the amino acid glycine and widely found by a variety of plants in nature. There is increasing evidence that it is a highly valuable feed additive and can produce positive effects on animal performance [7,8,9]. Betaine is known to have two major functions in the body, as a methyl group donor and an organic osmolyte. On the other hand, betaine has been shown to protect cells from osmotic pressure and allow them to continue normal metabolic activities under conditions that inactivate cells [10]; thus, the use of betaine may improve broiler tolerance to heat stress. Furthermore, it has been suggested that betaine could be used as a natural antioxidant and had the ability to improve meat quality of broilers [11]. Based on the above properties of betaine, previous studies have demonstrated that dietary betaine could improve the heat stressed broilers’ growth performance, physiology, carcass criteria [12], lipid metabolism [13], immune response [14], and intestinal barrier function [15]. However, the findings of one study were inconsistent [16], which revealed that betaine supplementation had no significant effects on carcass traits and intestinal morphology of broilers under heat stress. The variable results suggested that further research and development is still required in this regard. Meanwhile, due to the good meat quality, yellow-feathered broilers are increasingly favored by Chinese consumers. Huaixiang chicken is a famous Chinese indigenous yellow-feather broiler breed and is widely farmed in southern China [17]. However, there is extremely limited information about the effects of betaine on these indigenous yellow-feathered broilers under long-term heat stress. Therefore, the current experiment was conducted to investigate the adverse effect of long-term heat stress on growth performance, digestive function, carcass traits, and meat quality in indigenous yellow-feathered broilers (Huaixiang chicken), and to evaluate whether feeding betaine could ameliorate long-term heat stress-induced impairment of these parameters.

## 2. Materials and Methods

### 2.1. Animal Ethics

The present study was carried out at the College of Agriculture, Guangdong Ocean University, Zhanjiang, China. The protocol of this experiment was approved by the Animal Care Committee, College of Agriculture, Guangdong Ocean University, Zhanjiang, China (SYXK-2018-0147).

### 2.2. Experimental Design, Animals, and Diet

A total of two hundred and forty 5-week-old yellow-feathered male broilers (indigenous breed, Huaixiang chickens) were randomly allocated to five treatments, each of which was replicated six times with eight broilers per replicate. The experimental period lasted 10 weeks. The five treatments were thermoneutral zone control group (TN, fed basal diet); heat stress control group (HS, fed basal diet); heat stress treatment group 1 (basal diet +500 mg/kg betaine); heat stress treatment group 2 (basal diet +1000 mg/kg betaine); heat stress treatment group 3 (basal diet +2000 mg/kg betaine). The betaine was obtained from a commercial Chinese company (anhydrous betaine, 99% purity, Shandong Jianchuan Biotechnology Co., Ltd., Shandong, China). The ingredient composition and nutrient content of basal diets are presented in Table 1. Basal diets were formulated to meet or exceed requirements suggested by the Chinese Chicken Feeding Standard (NY/T33-2004) [18]. The diet was given to the birds in the form of mash, and betaine was mixed into the diet before feeding. To ensure that the betaine was thoroughly mixed into the diet, firstly, betaine was mixed with 1 kg of feed by hand, and then the premix was mixed with the remaining feed by using a blender. The crude protein, lysine, cystine, methionine, calcium, and phosphorus of the diet were determined according to the methods of AOAC (2000) [19]. Birds had free access to feed and water. The broilers in TN group were raised at 26 ± 1 °C during the whole study. Other groups, designed as HS groups, were subjected to cyclic heat stress by exposing them to 32 ± 1 °C for 8 h/day from 9:00 am to 17:00 pm, the temperature of rest time is consistent with TN groups. Relative humidity was controlled at 65–75% among all groups during the entire experimental period. The birds in the TN and HS groups were housed in different facilities, the temperature and relative humidity of the TN and HS groups were measured three times a day. Continuous artificial light was used to illuminate the interior space for the whole period. The chicken houses were equipped with environmental control equipment, and the size of cage is 90 (length) × 70 (width) × 40 (height) cm.

### 2.3. Sampling and Measurements

The cage was considered as the experimental unit. Broilers were weighed on a cage basis (*n* = 30) initially and after 5 and 10 weeks of heat stress. The feed consumption was recorded weekly based on the cage (*n* = 30). Body weight gains (BWG), feed intake, and feed conversion ratio (FCR) were then calculated using this information for each phase.

After 5 and 10 weeks of heat stress, one bird from each replicate was randomly selected and moved to metabolic cages for metabolic testing (one bird per cage). The metabolic test lasted 4 days, and the nutrient retention was analyzed as average data by cage during the 4 days (*n* = 30). The total excreta collection method was used for determination of nutrient retention. During the test, feed intake and excrements were recorded daily, and the excreta were collected. The nitrogen, ash, gross energy, Ca, and phosphorus contents in the feed and excreta were then analyzed based on the method of AOAC (2000) [19]. The crude fat contents were analyzed by using a fat analyzer (Hua Bei Experimenting, Co., Ltd., Hebei, China) based on the Soxhlet extraction method, and ether was used as the solvent. The nutrient retention was calculated by the following formula:

Nutrient retention (%) = (feed intake × Nf- excretion amount × Ne)/(feed intake × Nf) × 100

where Ne = nutrient concentration in excreta (% DM), Nf = nutrient concentration in feed (% DM).

After 5 and 10 weeks of heat stress, six birds per treatment (one bird per replicate was randomly selected) were slaughtered (*n* = 30) by severing the jugular vein, respectively. Small intestine was then separated and samples of the contents of the duodenum, jejunum, and ileum were immediately collected for the determination of digestive enzyme activity by using a commercial kit (Nanjing Jiancheng Bioengineering Institute, Nanjing, China). Subsequently, approximately 2 cm segments of the duodenum, jejunum, and ileum at the middle position were collected immediately. The intestinal samples from each section were fixed in 10% buffered formalin until analyzed. Each intestinal segment was embedded in paraffin. A 7 μm section of each sample was placed onto a glass slide and stained with alcian blue/haematoxylin and eosin for examination with a light microscope. Villus height and crypt depth of the small intestine were measured at 40× magnification using computer software (Sigma Scan, Jandel Scientific, San Rafael, CA, USA), then villus height to crypt depth ratio was calculated.

At the end of the experiment (after 10 weeks of feeding trial), the carcass traits and meat quality of the slaughtered broilers were determined (one bird per replicate was randomly selected, *n* = 30). The carcass traits, included: slaughter rate (%) = (slaughter weight/live weight) × 100; semi-eviscerated carcass rate (%) = (semi-eviscerated weight/live weight) × 100; eviscerated carcass rate (%) = (eviscerated weight/live weight) × 100; leg muscle yield (%) = (leg muscle weight on both sides/live weight) × 100; breast muscle yield (%) = (breast muscle weight on both sides/live weight) × 100; abdominal fat rate (%) = (abdominal fat weight/live weight) × 100. Subsequently, cooking loss was measured by using approximately 20 g of meat sample from the left breast and leg muscle according to the method described by Honikel [20]. The shear force of breast and leg muscle was detected by using C-LM3 digital display tenderness meter (kgf, Northeast Agricultural University, Harbin, China). Duplicate pH values of leg and breast muscle for each sample at 45 min and 24 h after slaughtered were measured using a pH meter (PHSJ-5, Leici, Shanghai Yidian Scientific Instrument Co., Ltd., Shanghai, China).

### 2.4. Statistical Analysis

All data were analyzed by using SAS 9.4 (SAS Institute Inc., Cary, NC, USA). The growth performance was analyzed during 1–5 weeks, 6–10 weeks, and 1–10 weeks of heat stress. Nutrient retention, digestive enzyme activity, and intestinal morphology were analyzed after 5 and 10 weeks of heat stress. Carcass traits and meat quality were investigated after 10 weeks of heat stress. Data were expressed as means. Differences among means were tested by using Tukey’s test. Orthogonal polynomial contrasts were used to test the linear, quadratic, and cubic effects of the increasing levels of dietary betaine among HS groups. Variability in data was expressed as standard error of means (SEM), *p* < 0.05 was considered to be statistically significant, 0.05 ≤ *p* < 0.10 was considered to be a tendency.

## 3. Results

### 3.1. Growth Performance

The results of growth performance were shown in Table 2. During 1–5 weeks, heat stress reduced the BWG and feed intake (*p* < 0.05), whereas it increased the FCR (*p* < 0.05). Dietary betaine supplementation tended to improve the BWG (linear, *p* = 0.078) and feed intake (linear, *p* = 0.075) of broilers under heat stress. During 6–10 weeks, heat stress decreased the BWG and feed intake (*p* < 0.01). Supplementation of graded levels of betaine improved the BWG and feed intake (linear, *p* < 0.05) of broilers under heat stress. During the whole experimental period (1–10 weeks), heat stress reduced the BWG and feed intake (*p* < 0.01) and dietary inclusion of betaine increased BWG and feed intake (linear, *p* < 0.05) of broilers under heat stress.

### 3.2. Nutrient Retention

Nitrogen retention was significantly reduced by 5 weeks of heat stress (Table 3, *p* < 0.05), and 5 weeks of heat stress tended to decrease the P retention (*p* = 0.065). Supplementation of betaine increased the nitrogen and P retention (linear, *p* < 0.05). After 10 weeks of heat stress, decreased nitrogen retention was observed (*p* < 0.05). Dietary betaine could improve nitrogen retention in heat stressed broilers (linear, *p* < 0.05).

### 3.3. Digestive Enzyme Activity

As presented in Table 4, after 5 weeks of heat stress, the trypsin activity of the jejunum was decreased by heat stress (*p* < 0.05). Detary supplementation of betaine had quadratic effects on trypsin activity of jejunum in heat stressed broilers (*p* < 0.05). Additionally, after 10 weeks of heat stress, dietary betaine supplementation improved the trypsin activity of the duodenum in heat stressed broilers (linear, *p* < 0.05).

### 3.4. Intestinal Morphology

**Figure 1:**
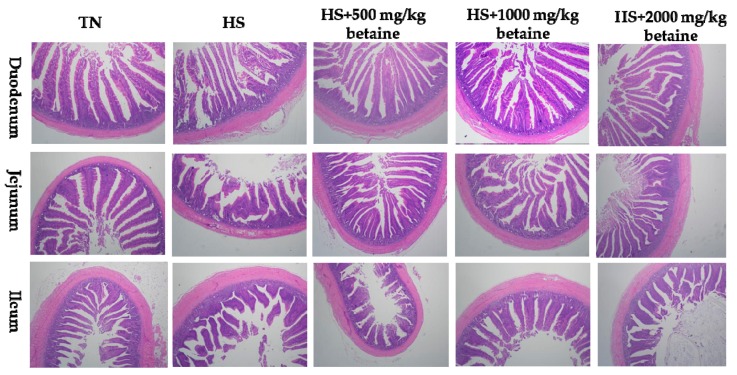
Photomicrographs of the effects of dietary betaine on intestinal morphology of yellow-feathered broilers after 5 weeks of heat stress (Stained with hematoxylin and eosin; TN, thermoneutral zone; HS, heat stress).

**Figure 2:**
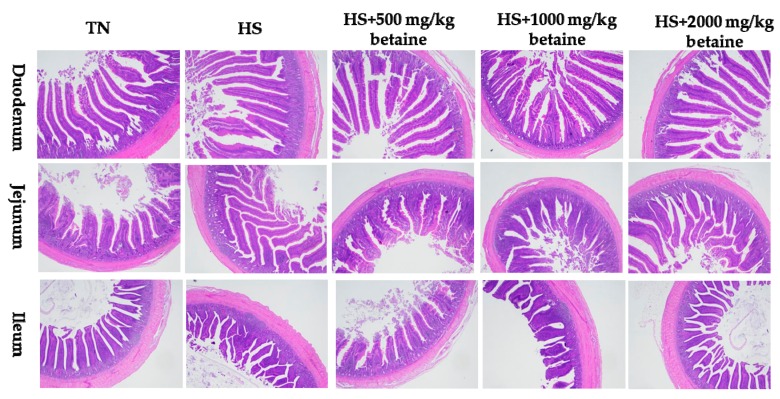
Photomicrographs of the effects of dietary betaine on intestinal morphology of yellow-feathered broilers after 10 weeks of heat stress (Stained with hematoxylin and eosin; TN, thermoneutral zone; HS, heat stress).

As shown in Table 5 and Figure 1 and Figure 2, after 5 weeks of heat stress, as compared with the TN group, the heat stress control group had lower villus height (*p* < 0.05) and tended to decrease the villus height to crypt depth ratio of duodenum (*p* = 0.057). Supplemental betaine had a tendency to increase the villus height to crypt depth ratio (linear, *p* = 0.057), and had a trend of quadratic effect on villus height (*p* = 0.084) and villus height to crypt depth ratio (*p* = 0.056) of the duodenum in heat-stressed treatments. After 10 weeks of the feeding trial, heat stress induced reduction of villus height of the duodenum and jejunum (*p* < 0.05), and decreased the villus height to crypt depth ratio of the jejunum (*p* < 0.05). Supplementation with betaine improved the villus height and villus height to crypt depth ratio of the jejunum (linear, *p* < 0.05) and had quadratic effects on villus height and villus height to crypt depth ratio of the duodenum (*p* < 0.05) in heat stressed broilers.

### 3.5. Carcass Traits

After 10 weeks of heat stress, broilers in the heat stress control group had lower eviscerated carcass rate and breast muscle yield than those in TN group (Table 6, *p* < 0.05). Additionally, heat stress tended to reduce semi-eviscerated carcass rate (*p* = 0.085). Dietary betaine improved the semi-eviscerated carcass rate, eviscerated carcass rate, and breast muscle yield of heat stressed broilers (linear, *p* < 0.05).

### 3.6. Meat Quality

The results of meat quality were presented in Table 7. Heat stress challenge had no effects on cooking loss, shear force, and pH of breast and leg muscle (*p* > 0.05). In heat stress treatments, dietary betaine supplementation had no significant effects on investigated meat quality parameters (*p* > 0.05).

## 4. Discussion

### 4.1. Growth Performance

It has been well documented that heat stress causes a series of drastic changes in broilers’ physiological function, including decreasing the feed intake, disturbing the intestinal function and electrolyte balance, and adversely affecting blood metabolites and hormonal secretions, which results in impairment of productive performance [2,4]. In this study, expectedly, heat stress induced a reduction in BWG and feed intake of indigenous yellow-feathered broilers. This was in agreement with the reports by Zhong et al. [21,22], who observed that heat stress suppressed the average daily gain of yellow-feathered broilers under similar experimental conditions. The lower growth rate in heat stressed broilers may be attributed to the decreased feed intake, which is a defense mechanism to reduce the heat increment of bodies [23]. In addition, the heat stressed broilers consume more energy to adapt to high ambient temperature, thereby reducing energy for growth and leading to a lower growth performance [1].

Betaine is a functional active substance from a variety of plants, which can act as methyl group donor and organic osmolyte, and has the ability to improve growth performance in animals [7,8,9]. Meanwhile, according to the previous studies, betaine could be used as an effective antistress additive in broilers. For instance, He et al. [13] demonstrated that betaine improved the BWG and feed intake of Arbor Acres broilers under 32 °C heat stress. Chand et al. [14] found that dietary supplementation of 1.5% and 2% betaine increased the feed intake and BWG and reduced the FCR of fast-growing broilers exposed to heat stress. Similar findings have been reported in the studies of Sakomura et al. [16] and Singh et al. [24], who observed a significant increase in feed intake and BWG of heat stressed Cobb broilers fed with diet contained betaine. However, the studies related to the effects of betaine on indigenous slow-growing broilers are very scarce. Attia et al. [12] reported that 0.5 or 1.0 g/kg betaine supplementation improved the BWG and feed intake, whereas it decreased the FCR of slow-growing white-feathered broilers under heat stress. To the best of our knowledge, no research has been reported to study the effect of dietary betaine on Chinese indigenous yellow-feathered broilers. Our data showed that dietary supplementation of betaine could mitigate the adverse effects of heat stress on feed intake and BWG in indigenous yellow-feathered broilers, indicating that betaine has potential as an anti-heat stress additive for slow-growing yellow-feathered broilers. Regarding the mechanism of action, it was assumed that the beneficial effects on growth performance of heat stressed broilers might be due to the osmoregulatory, methyl group donors, and antioxidative properties of betaine.

### 4.2. Digestive Function

During heat stress, the intestinal epithelial cells of broilers are subjected to osmotic stress, as high ambient temperature may lead to water imbalance and cell permeability changes through dehydration [25]. Additionally, the fluid transport in the gastrointestinal tract during heat stress may also cause changes in intestinal structure and digestive function [23]. Indeed, in the current study, heat stress groups had lower nitrogen retention and trypsin activity. This was supported by previous findings of Attia et al. [12] and Chen et al. [26], who demonstrated that heat stress decreased the nitrogen digestibility and digestive enzymes activity of broilers. The present study showed that dietary supplementation of betaine ameliorated the nitrogen retention and trypsin activity of the duodenum and jejunum in heat stressed yellow feather broilers. Similarly, Attia et al. [12] reported that 0.5 or 1.0 g/kg betaine supplementation recovered the crude protein digestibility coefficients from the adverse effects of heat stress on slow-growing chicks. Eklund et al. [27] revealed that supplementation of betaine in broilers’ diet could improve the apparent nutrient digestibility, including protein, methionine, and crude fat. However, because the available data regarding the effect of betaine on nutrient digestibility and digestive enzymes activity in heat stressed yellow-feathered broilers is limited, no more comparisons could be made. On the other hand, according to the results obtained by Wang et al. [28], betaine supplementation increased the activities of amylase, lipase, trypsin, and chymotryps of the small intestine in stressed rats. Pollard and Wyn Jones [29] also found that betaine protected against stress inhibition of enzymes. It has been suggested that betaine could promote the activity of key cellular enzymes, and the effects of betaine involved universal water–solute–macromolecule interactions [28]. Betaine possesses an osmotic effect and attaches to the surface of biopolymers and helps proteins fold more compactly [30]. In the same study, they also noted that this protective effect may be limited to certain enzymes. This was in agreement with our results, which only show an increase in the activity of trypsin.

The intestinal villus height and villus height to crypt depth ratio were decreased by heat stress in our experiment, suggesting that heat stress induced deterioration of intestinal morphology. These findings were in accordance with previous reports of Quinteiro-Filho et al. [2] and Burkholder et al. [3]. Animals have mechanisms to regulate body temperature as well as changes in physiological status. When the ambient temperature exceeds the thermoneutral zone, the body temperature raises, and peripheral blood flow increases as a response to heat stress, meaning that the blood flow of turbinate, nasal mucosa, myocardium, and respiratory muscles is higher than that of the intestine [2]. Ischemia and hypoxia of the intestine can cause epithelial shedding, leading to a deeper crypt depth and shorter villus height [3]. This study showed that the intestinal epithelial morphology was revived by the inclusion of betaine. Several possible mechanisms could explain the positive response of heat stressed broilers to dietary betaine: the methyl group donor nature of betaine might promote the proliferation of intestinal epithelial cells; the osmotic effect of betaine could improve the intestinal environment; and the antioxidant activity of betaine could alleviate intestinal oxidative damage induced by heat stress [28]. However, most studies to date have only investigated the effects of betaine on intestinal morphology of fast-growing broilers or rats. For instance, Kettunen et al. [31] discovered that dietary betaine supplementation increased the villus-crypt ratio of intestine in broilers. Eklund et al. [27] reported that betaine could maintain gut villi integrity and consequently promote better nutrient digestibility and absorption in broilers. Wang et al. [28] also demonstrated that betaine supplementation enhanced villus heights and villus height to crypt depth ratio of the duodenum, jejunum, and ileum in stressed rats. One study on the effects of betaine on heat stressed broilers obtained a contrary finding [16], revealing that the morphometrics of the intestinal crypts and villi in heat stressed broilers were not influenced by supplementation of betaine. The extent and duration of heat stress, species of broilers, growth stages, and the type of diet could help to explain these inconsistencies. Overall, betaine favorably affected the intestinal structure and digestive function could account for the boosted growth performance in this study.

### 4.3. Carcass Traits and Meat Quality

The present study discovered that the eviscerated carcass rate and breast muscle yield were reduced in response to heat exposure. This was supported by previous findings of Lu et al. [32], who reported that the carcass parameters were negatively affected by chronic heat stress in broilers. However, Sakomura et al. [16] did not find any significant impacts of heat stress on carcass, leg, and breast yield. The possible reasons of these results might be due to the experimental conditions and genetic background of broilers. In that study, thermoneutral zone groups were held at 28 °C from day 22 to day 45, and they used fast-growing (Cobb) broilers; these differences could lead to inconsistent findings. Betaine is often considered as a carcass modifier due to methyl group donor property, which causes a higher availability of methionine and cystine for protein deposition, thus contributing to improving the carcass lean percentage [33]. In this study, when supplemented with betaine in heat stressed broiler groups, the carcass traits were subsequently improved. Consistent with our results, Attia et al. [12] observed an improvement in the carcass traits of heat stressed slow-growing chicks by dietary betaine. Nofal et al. [34] found that inclusion of 0.2% betaine increased carcass weight and breast muscle yield in growing broilers under heat stress conditions. Similar results in thermoneutral conditions were obtained by Rao et al. [7] and Zhan et al. [35], who reported that dietary betaine supplementation enhanced the breast muscle yield of male broiler chickens.

Regarding meat quality, even though some studies have suggested that chronic heat stress had adverse effects on the meat quality of broilers, such as drop loss, cooking loss, shear force, pH, and meat color [4,5,6], our study failed to show any significant impacts of heat stress on cooking loss, shear force, or pH of breast and leg muscle. The different broiler breeds used might explain the difference of these results. Feeding betaine ameliorated heat stress-induced impairment of meat quality according to Attia et al. [12], who suggested that dietary betaine improved dry matter composition and water holding capacity of meat in slow-growing broilers. Also, Alirezaei et al. [11] indicated that betaine could act as an antioxidant agent and improve broilers’ meat quality. However, there were no significant differences in meat quality criteria between treatments in this study. This might be because the number of observations was insufficient, or the detected criteria of meat quality were limited. Increasing the sample size of the experiment, and investigating other criteria related to meat quality, such as TBARS, intramuscular fat, lactic acid, etc., are essential in future studies.

## 5. Conclusions

To summarize, the current results indicated that long-term heat stress induced inferior growth performance, injured digestive function, and lower carcass yield in indigenous yellow-feathered broilers. Dietary supplementation of betaine was effective in improving growth performance, digestive function, and carcass traits in indigenous yellow-feathered broilers subjected to heat stress.

## Figures and Tables

**Table 1:** Basal diet composition (as-fed basis).

Item	Contents (%)
Ingredients	
Corn	67
Soybean meal	23
Wheat bran	4.0
Fish meal	3.0
Limestone	1.5
CaHPO_4_	1.0
Premix ^1^	0.5
Nutrient levels ^2^	
ME (MJ/kg)	11.94
Crude protein (%)	18.2
Ca (%)	0.98
Met (%)	0.32
Cystine (%)	0.31
Lys (%)	0.90
Total phosphorus (%)	0.51

^1^ Premix provided per kilogram of diet: 5000 IU of vitamin A, 1000 IU of vitamin D_3_, 10 IU of vitamin E, 0.5 mg of vitamin K_3_, 3 mg of thiamin, 7.5 mg of riboflavin, 4.5 mg of vitamin B_6_, 10 μg of vitamin B_12_, 25 mg of niacin, 0.55 mg of folic acid, 0.2 mg of biotin, 500 mg of choline, and 10.5 mg of pantothenic acid. 60 mg of Zn, 80 mg of Mn, 80 mg of Fe, 3.75 mg of Cu, and 0.35 mg of I. ^2^ Nutrient levels on DM basis; except for metabolic energy (ME), others are measured values; ME calculated according to Chinese feed ingredient database.

**Table 2:** Effects of heat stress and dietary betaine on growth performance of yellow-feathered broilers *.

Dietary Betaine Levels (mg/kg)	Temperature	Initial BW, g	1–5 Weeks			6–10 Weeks			1–10 Weeks		
			BWG, g	FI, g	FCR	BWG, g	FI, g	FCR	BWG, g	FI, g	FCR
0	TN	404	426	1441	3.42	459	1729	3.88	992	3170	3.22
0	HS	393	328	1345	4.14	299	1248	4.28	720	2593	3.69
500	HS	399	369	1312	3.59	424	1575	3.84	849	2887	3.46
1000	HS	406	371	1429	3.88	363	1616	4.60	828	3046	3.70
2000	HS	391	385	1404	3.81	449	1671	3.66	939	3075	3.30
SEM		9.9	20.5	30.4	0.187	35.9	83.1	0.362	57.9	85.5	0.214
Contrast			*p*-value								
TN vs. HS		-	0.004	0.042	0.013	0.005	0.001	0.435	0.004	0.001	0.142
Linear		-	0.078	0.075	0.468	0.024	0.037	0.236	0.042	0.026	0.276
Quadratic		-	0.581	0.917	0.279	0.581	0.516	0.288	0.885	0.222	0.589

* BW, body weight; BWG, body weight gain; FI, feed intake; FCR, feed conversion ratio; TN, thermoneutral zone; HS, heat stress; SEM, standard error of means; TN vs. HS, TN group vs. HS control (0 mg/kg betaine) group.

**Table 3:** Effects of heat stress and dietary betaine on nutrient retention of yellow-feathered broilers *, %.

Dietary Betaine Levels (mg/kg)	Temperature	Nitrogen	CF	Energy	Ash	Ca	P
**After 5 Weeks Heat Stress**							
0	TN	79	86	77	47	59	50
0	HS	70	83	79	45	61	37
500	HS	77	84	81	47	57	46
1000	HS	79	80	79	54	63	55
2000	HS	79	85	82	58	62	52
SEM		2.7	3.1	2.3	5.1	4.3	4.7
Contrast		*p*-value					
TN vs. HS		0.032	0.579	0.554	0.786	0.843	0.065
Linear		0.027	0.932	0.486	0.092	0.686	0.045
Quadratic		0.215	0.419	0.905	0.828	0.828	0.250
**After 10 Weeks Heat Stress**							
0	TN	70	84	82	42	60	50
0	HS	64	85	81	45	59	43
500	HS	69	84	84	46	58	42
1000	HS	66	82	81	52	57	51
2000	HS	70	85	79	50	65	47
SEM		1.5	2.8	2.4	4.1	3.5	3.3
Contrast		*p*-value					
TN vs. HS		0.019	0.808	0.619	0.576	0.817	0.178
Linear		0.046	0.894	0.894	0.644	0.393	0.271
Quadratic		0.740	0.428	0.428	0.711	0.287	0.741

* CF, crude fat; TN, thermoneutral zone; HS, heat stress; SEM, standard error of means; TN vs. HS, TN group vs. HS control (0 mg/kg betaine) group.

**Table 4:** Effects of heat stress and dietary betaine on digestive enzyme activity of yellow-feathered broilers*, U/mg protein.

Dietary Betaine Levels (mg/kg)	Temperature	Duodenum		Jejunum		Ileum	
		Trypsin	Lipase	Trypsin	Lipase	Trypsin	Lipase
**After 5 Weeks Heat Stress**							
0	TN	248	1.76	351	1.69	346	1.71
0	HS	179	1.30	247	1.33	315	1.63
500	HS	172	1.72	361	2.68	380	2.25
1000	HS	196	1.05	310	1.36	335	1.65
2000	HS	189	1.36	271	1.40	333	1.55
SEM		33.1	0.518	32.6	0.491	54.0	0.462
Contrast		*p*-value					
TN vs. HS		0.151	0.537	0.035	0.605	0.686	0.904
Linear		0.590	0.845	0.898	0.653	0.975	0.695
Quadratic		1.000	0.926	0.038	0.239	0.559	0.461
**After 10 Weeks Heat Stress**							
0	TN	225	0.89	414	1.61	293	0.64
0	HS	116	0.55	313	0.91	243	0.60
500	HS	183	1.23	264	1.31	216	0.50
1000	HS	201	0.43	418	2.35	293	0.54
2000	HS	250	0.99	258	1.85	263	0.81
SEM		40.4	0.382	56.7	0.703	41.3	0.178
Contrast		*p*-value					
TN vs. HS		0.105	0.528	0.296	0.488	0.406	0.865
Linear		0.044	0.767	0.972	0.249	0.480	0.287
Quadratic		0.849	0.879	0.327	0.539	0.978	0.339

* TN, thermoneutral zone; HS, heat stress; SEM, standard error of means; TN vs. HS, TN group vs. HS control (0 mg/kg betaine) group.

**Table 5:** Effects of heat stress and dietary betaine on intestinal morphology of yellow-feathered broilers *.

Dietary Betaine Levels (mg/kg)	Temperature	Duodenum			Jejunum			Ileum		
		Villus Height, μm	Crypt Depth, μm	VH:CD	Villus Height, μm	Crypt Depth, μm	VH:CD	Villus Height, μm	Crypt Depth, μm	VH:CD
**After 5 Weeks Heat Stress**										
0	TN	523	65	8.09	397	49	8.25	215	45	4.83
0	HS	415	68	6.14	335	46	7.28	204	42	4.99
500	HS	474	63	7.68	337	49	7.02	233	42	5.58
1000	HS	559	60	9.50	330	48	7.12	248	50	4.94
2000	HS	479	64	7.88	338	53	6.43	231	47	5.00
SEM		34.7	3.9	0.679	25.8	3.1	0.803	21.9	4.0	0.352
Contrast		*p*-value								
TN vs. HS		0.039	0.599	0.057	0.102	0.511	0.403	0.728	0.605	0.745
Linear		0.122	0.387	0.057	0.983	0.162	0.456	0.340	0.165	0.708
Quadratic		0.084	0.253	0.056	0.918	0.706	0.773	0.299	0.586	0.483
**After 10 Weeks Heat Stress**										
0	TN	566	105	5.54	318	66	4.95	192	59	3.26
0	HS	480	106	4.78	212	68	3.27	177	58	3.16
500	HS	540	87	6.42	265	60	4.55	228	58	4.15
1000	HS	555	102	5.84	287	63	4.72	209	58	3.60
2000	HS	529	109	5.03	283	61	4.99	189	60	3.13
SEM		22.9	8.4	0.508	21.9	6.0	0.434	23.9	5.9	0.378
Contrast		*p*-value								
TN vs. HS		0.036	0.975	0.370	0.003	0.781	0.028	0.664	0.885	0.850
Linear		0.114	0.541	0.751	0.037	0.405	0.037	0.882	0.822	0.717
Quadratic		0.046	0.155	0.033	0.232	0.640	0.347	0.179	0.898	0.080

* VH:CD, villus height to crypt depth ratio; TN, thermoneutral zone; HS, heat stress; SEM, standard error of means; TN vs. HS, TN group vs. HS control (0 mg/kg betaine) group.

**Table 6:** Effects of heat stress and dietary betaine on carcass traits of yellow-feathered broilers *, %.

Dietary Betaine Levels (mg/kg)	Temperature	Slaughter Rate	Semi-Eviscerated Carcass Rate	Eviscerated Carcass Rate	Leg Muscle Yield	Breast Muscle Yield	Abdominal Fat Rate
0	TN	92.5	85.0	61.5	14.0	8.5	1.05
0	HS	91.0	83.3	58.5	14.6	7.4	1.47
500	HS	91.8	84.4	60.0	14.1	8.3	1.05
1000	HS	90.7	84.0	59.3	15.2	8.3	1.10
2000	HS	92.3	85.4	62.9	15.7	8.7	1.29
SEM		0.82	0.66	0.99	0.80	0.33	0.249
Contrast		*p*-value					
TN vs. HS		0.197	0.085	0.044	0.575	0.047	0.255
Linear		0.511	0.048	0.019	0.223	0.014	0.702
Quadratic		0.662	0.592	0.328	0.485	0.956	0.304

* TN, thermoneutral zone; HS, heat stress; SEM, standard error of means; TN vs. HS, TN group vs. HS control (0 mg/kg betaine) group.

**Table 7:** Effects of heat stress and dietary betaine on meat quality of yellow-feathered broilers *.

Dietary Betaine Levels (mg/kg)	Temperature	Breast Muscle				Leg Muscle			
		Cooking Loss, %	Shear Force, kgf	pH45min	pH24h	Cooking Loss, %	Shear Force, kgf	pH45min	pH24h
0	TN	38.2	2.83	5.75	5.30	40.3	1.99	5.78	5.71
0	HS	36.3	2.92	5.59	5.37	38.2	1.85	5.82	5.47
500	HS	32.5	2.46	5.87	5.55	35.0	1.55	5.76	5.64
1000	HS	38.4	2.20	5.74	5.45	39.1	1.72	6.00	5.50
2000	HS	34.8	2.77	5.58	5.45	38.4	2.12	5.97	5.40
SEM		2.51	0.259	0.143	0.130	2.15	0.264	0.102	0.139
Contrast		*p*-value							
TN vs. HS		0.545	0.809	0.726	0.413	0.509	0.698	0.233	0.818
Linear		0.913	0.521	0.773	0.802	0.618	0.438	0.153	0.605
Quadratic		0.969	0.052	0.107	0.522	0.558	0.215	0.930	0.396

* TN, thermoneutral zone; HS, heat stress; SEM, standard error of means; TN vs. HS, TN group vs. HS control (0 mg/kg betaine) group.
